# New Insights About Doxorubicin-Induced Toxicity to Cardiomyoblast-Derived H9C2 Cells and Dexrazoxane Cytoprotective Effect: Contribution of *In Vitro* ^1^H-NMR Metabonomics

**DOI:** 10.3389/fphar.2020.00079

**Published:** 2020-02-20

**Authors:** Matthieu Dallons, Corentin Schepkens, Aurélie Dupuis, Vanessa Tagliatti, Jean-Marie Colet

**Affiliations:** Department of Human Biology and Toxicology, Faculty of Medicine and Pharmacy, University of Mons, Mons, Belgium

**Keywords:** doxorubicin, cardiotoxicity, dexrazoxane, H9C2, metabonomics, ^1^H-NMR

## Abstract

Doxorubicin (DOX) is an anticancer drug widely used in oncology. The main limitation to DOX treatments though is due to the cumulative dose that may lead to cardiotoxicity. Clinically, DOX-induced cardiomyopathy develops as a progressive heart failure consecutive to a progressive loss in cardiomyocytes due to cell necrosis and apoptosis induced by DOX. For many years, the cardiac oxidative stress caused by DOX was considered as its main toxic mechanism. Therefore, several clinical trials were carried out to assess the efficacy of various antioxidants as a cardioprotective strategy. Only dexrazoxane (DEX), did significantly reduce DOX cardiotoxicity. However, since other antioxidants used later on to counteract DOX cardiotoxicity were not as successful as DEX, DOX-induced oxidative stress and DEX antioxidant activity are not considered as the main feature anymore and this led the scientific world to suspect other involved mechanisms which are still unknown. The objective of the present work was to study from a metabolic point of view the side effects of DOX and the protective properties of DEX. *In vitro* ^1^H-NMR metabonomics was applied to the rat cardiomyoblastic H9C2 cell line. This strategy was used with the hope of unveiling possible new targets to cope with DOX cardiotoxicity. Another underlying goal was the validation of H9C2 *in vitro* model for metabolic investigations of DOX and DEX effects. For this purpose, several parameters, including oxidative stress, cell mortality, and apoptosis, were measured to assess the effects of DOX and DEX alone or in combination. The metabonomic study was carried out on cellular fluids collected after either 4 or 24 hours of DOX-exposure. Under such experimental conditions, both the major adverse effects reported in patients exposed to DOX and the protective effect of DEX were demonstrated *in vitro,* suggesting that the H9C2 *in vitro* model is relevant to investigate both DOX cardiotoxicity and putative cardioprotective strategies. In addition, the metabonomics findings highlighted several metabolic pathways involved in DOX cardiotoxicity and DEX cardioprotective effects as potential metabolic targets for cardioprotection: energy metabolism, redox balance, as well as phospholipids and proteins metabolism.

## Introduction

Doxorubicin (DOX), belonging to the anthracyclines family, is a widely used anti-cancer drug employed among others in the treatment of breast cancer, sarcomas, and hematologic tumors. Currently, DOX anti-cancer treatment is limited by its puzzling cardiotoxicity (Octavia et al., 2012; Chung and Youn, 2016). The severity and irreversibility of the toxicity depend on cumulated DOX doses and can range from subclinical myopathy to patient's death in the worst cases (Groarke and Nohria, 2015). Incidences of DOX cardiotoxicity step from 5% for a cumulative dose of 400 mg/m^2^ to 26% for 550 mg/m^2^ (recommended as the maximum cumulative dose for a therapy). The main toxicological mechanism involved in this cardiotoxicity is the surproduction of reactive oxygen species (ROS) through two intracellular pathways. The first pathway involves the quinone functional group of the DOX molecule. This quinone can undergo a redox cycle initiated by different enzymes such as nicotinamide adenine dinucleotide phosphate (NADPH) oxidases and mitochondrial complex I. The semiquinone group in turn reacts with oxygen to produce superoxide anion. The second pathway is iron-dependent. The anthracycline also binds iron through its quinone and semiquinone chemical groups, leading to the synthesis of hydroxyl radical, an highly reactive ROS (Langer, 2014; Chung and Youn, 2016; Mitry and Edwards, 2016). The so-formed ROS promote an oxidative stress impairing mitochondrial function and membranes integrity (Langer, 2014). This results in cardiomyocytes death by apoptosis and necrosis, clinically expressed by a progressive heart failure (Damiani et al., 2016).

Nowadays, the main primary strategy to deal with this cardiotoxicity is the co-administration of a cardioprotective agent counteracting the oxidative stress (Chung and Youn, 2016). The only approved cardioprotective agent is dexrazoxane (DEX), acting by reducing ROS production through iron chelation. DEX is intravenously injected to patients 10 to 30 minutes. prior the onset of DOX treatment, at a recommended DEX/DOX dose ratio of 10:1 (Langer, 2014). Clinically, DEX cardioprotective effect has been studied for more than 20 years. There are evidences that the incidence of heart failure is significantly reduced in patients pre-treated with DEX. In 2013, a review of the clinical results from 12 randomized trials and two observational studies involving patients with no prior history of heart failure was published (Kalam and Marwick, 2013). It showed a significant decrease of cardiac events for patients pre-treated with DEX with a risk ratio of 0.35 [RR=0.35 (95% CI 0.27–0.45), p < 0.00001].

Despite its clear capability to reduce the incidence of heart complications in DOX-treated cancerous patients, DEX can be responsible for different adverse effects. It has been reported in early-phase clinical studies that DEX can cause a reversible elevation of hepatic transaminases as well as some myelotoxicity (neutropenia and thrombocytopenia), limiting the dose given to the patient (Langer, 2014). More recently, the risk of long-term effects of DEX became a concern, particularly in pediatric patients. A threefold increase in the incidence of primary hematological malignancies was demonstrated in DEX-treated pediatric patients in two randomized studies. These findings led to a restriction of the indication of DEX for cardioprotection (Tebbi et al., 2007; Langer, 2014). Therefore, there is still a need for new cardioprotective strategies, hopefully safer and more efficacious than DEX.

Although the increased oxidative stress is considered as the main toxicological mechanism responsible of DOX cardiotoxicity, it remains unclear why, among all the antioxidants clinically tested, only DEX showed some substantial benefice. These unexpected findings led to the conclusion that the oxidative stress was most likely not the only toxic mechanism and other causes should be investigated. Besides the beneficial effect of DEX on DOX-induced oxidative stress, some authors reported that DEX is also able to prevent its inhibition on the mitochondrial topoisomerase IIβ (Sawyer, 2013), another considered cardiotoxicological mechanism (Zhang et al., 2012). This element contributes to claim that the oxidative stress and its prevention should not be considered anymore as the key events in DOX induced-cardiomyopathy and that other mechanisms should be further investigated. Since mitochondrial impairment is a major finding in DOX-induced cardiotoxicity, we decided to study its associated metabolic alterations, as well as the protective role of DEX, by a ^1^H-NMR metabonomic approach. Metabonomics should help unveiling new cellular targets to counteract DOX cardiotoxicity and to improve our understanding of DOX and DEX mode of actions.

## Materials and Methods

### Materials

The H9C2 (2-1) (ECACC 88092904) cell line was acquired from European Collection of Authenticated Cell Cultures (Salisbury, United Kingdom). Dulbecco Modified Eagle Medium (DMEM), L-glutamine, penicillin/streptomycin, and trypsin-EDTA 0.05% were obtained from Gibco (Thermo Fisher Scientific, Inc., Waltham, MA, USA). Fetal bovine serum (FBS), phosphate buffer solution (PBS), Dulbecco's phosphate buffer solution (D-PBS), Doxorubicin hydrochloride (DOX), dexrazoxane (DEX), glutaraldehyde, violet crystal, triton, and dichloro-dihydro-fluorescein diacetate (DCFH-DA) were purchased from Sigma-Aldrich (Saint-Louis, MO, USA).

### Cell Culture

H9C2 rat cardiomyoblasts were cultured in DMEM high glucose supplemented with heat-inactivated FBS 10%, L-glutamine 2 mM, antibiotics (100 unit/ml of penicillin and 100 µg/ml of streptomycin), incubated at 37°C and 5% CO_2_, in a humidified atmosphere. Culture medium was changed every 2–3 days and cells were splitted at 80% of confluence using Trypsin-EDTA 0.05%. For all exposure procedures, cells were first seeded in culture plates or flasks with a seeding density of 30.000 cells/cm^2^ and were kept growing during 48 hours before any exposure.

### Cell Viability Assay

The effects of DOX and DEX on H9C2 cell viability were evaluated by crystal violet assay on 96 wells plate. After exposing the cells to different doses of DOX and DEX, the culture medium was removed and the cellular layer washed twice with PBS. One hundred microliter of glutaraldehyde 1% (Sigma Aldrich) was added for 15 minutes at room temperature, then removed before adding 100 µl of violet crystal 1% for 30 minutes at room temperature. The plate was then amply washed under water flow and dried at room temperature. One hundred microliter of triton 0.2% was added and the plate was agitated for 60 minutes. The absorbance was read at 570 nm, using a VersaMax™ (Molecular Devices^®^) plate reader. Relative mean cellular viability was finally determined for each condition. These effects of DOX increasing doses on cellular viability were measured after 4 and 24 hours of incubation. Protective effect of DEX was measured for both 4 and 24 hours. For 4 hour exposure, the doses of DOX and DEX were respectively 5 and 50 µM. For the 24 hour exposure, the doses were respectively 0.3 and 3 µM.

### Caspase 3 Activity Assay

The apoptotic activity of DOX and DEX was indirectly evaluated by measuring the caspase 3 activity. H9C2 cells were splitted into four conditions: control group (n=6), 5 µM DOX exposed group (n=6), 50 µM DEX exposed group (n=6), and DEX pre-exposed group before DOX exposure (n=6). After 4 hour exposure, the cell medium was removed and the cellular layer was washed twice with PBS. Cells were then collected in PBS by scraping, and centrifuged at 260 G during 5 minutes at 4°C. Cell pellet was stored at −80°C for further analysis. Caspase 3 activity was measured using EnzChek^®^ Caspase-3 Assay Kit 1 (ThermoFisher Scientific), according to the manufacturer's instructions. Fluorescence intensity was read with a Glomax^®^ Explorer (Promega™) plate reader at 342/441 nm (E/E). Relative mean caspase 3 activity was determined for each condition.

### Oxidative Stress Quantification Assay

DOX-induced oxidative stress and protective effect of DEX in H9C2 cells were indirectly evaluated by measuring ROS production with a DCFH-DA fluorescence probe (Kalyanaraman et al., 2012). Cells were randomly assigned to groups and were exposed once to different doses of DOX in PBS during 2 hours, in 96 wells plate. Cells were also exposed once to different doses of DEX, keeping a DEX/DOX ratio of 10. A 10 mM H_2_O_2_ exposure was used as positive control and a negative control was made with non-exposed cells. After 2 hours of incubation, a 200 µM DCFH-DA solution (in DMSO) was added in each well to reach a final concentration of 100 µM. Cells were incubated during 30 minutes at 37°C and in darkness. Fluorescence intensity was read with a Glomax^®^ Explorer (Promega™) plate reader at 490/510 nm (E/E). The relative mean ROS concentration was determined for each condition.

### Cells Exposure and Samples Collection for ^1^H-NMR-Based Metabonomic Study

For metabonomic investigations, cells were randomly assigned into four groups: a control group (CTR) exposed only to vehicle, a DOX-exposed group (DOX), a DEX-exposed group (DEX), and a group pre-incubated with DEX during 30 minutes before adding DOX (DEX-DOX). For this metabonomic study, H9C2 cells were cultured in T-175 flasks and exposed to the molecules for either 4 or 24 hours. For the 4 hour protocol, selected doses were 5 µM for DOX and 50 µM for DEX. For the 24 hours timeframe, doses were 0.3 µM for DOX and 3 µM for DEX. After both times of incubation, culture medium was collected and stored at −80°C. Then, cells were washed twice with PBS and collected in 6 ml of cold methanol by scrapping and quickly frozen in liquid nitrogen before storage at −80°C (Martineau et al., 2011).

### Samples Preparation for ^1^H-NMR, Spectra Acquisition and Treatment

A chloroform-methanol-water extraction was applied to the collected cells to extract intracellular metabolites (Sapcariu et al., 2014). Briefly, cells were lysed by sonication and the addition of the three solvents allowed separation of cells content into two phases: a chloroform phase containing lipophilic metabolites and a methanol-water phase containing hydrophilic metabolites. The methanol-water phase was collected and evaporated with a speed vacuum. Polar metabolites were then dissolved into 700 µl of phosphate buffer (0.2 M Na_2_HPO_4_/0,04 M NaH_2_PO_4_, pH 7.4) and centrifuged at 10.000G during 10 minutes 650 µl of supernatant were mixed to 50 µl of trimethylsilylpropanoic acid (TSP) 3.5 mM in 5 mm diameter NMR tubes. For culture medium, 500 µl of medium were mixed to 250 µl of phosphate buffer and centrifuged at 10.000G during 10 minutes 650 µl of supernatant were mixed to 50 µl of TSP 3.5 mM in 5 mm diameter NMR tubes.

^1^H-NMR spectra of methanol-water phases and extracellular fluids were acquired by a Bruker Avance 500.16 MHz spectrometer with a 5 mm PABBO BB- probe. A NOESYPRESAT-1D sequence was used with 256 scans. The acquired FIDs were Fourier-transformed to obtain spectra which were baseline- and phase-corrected using MestReNova 11 (Mestrelab Research, Santiago de Compostela, Spain). The resonance arising from TSP was arbitrarily set to 0.00 ppm for further spectral calibration. Spectral area from 0.08 to 10 ppm was subdivided into sub-regions of 0,04 ppm width. Each subregion was then integrated. The spectral area from 4.20 to 5.32 ppm, corresponding to water peak, was suppressed and each subregion integral was normalized to the spectral total area, using Excel functionalities (Microsoft^®^). Data were imported into SIMCA P+ 12 (Umetrics^®^) for multivariate data analysis purpose.

### Multivariate Data Analysis, Metabolites Identification, and Statistical Tests

First, the binned and integrated data were submitted to principal component analysis (PCA), a non-supervised approach. Data were centered and R^2^_cum_ and Q^2^_cum_ parameters were determined. Then, data were projected to latent structure discriminant analysis (PLS-DA), a supervised method where the classes were defined as exposure groups. R^2^_cum_ and Q^2^_cum_ parameters as well as p-value of CV-ANOVA were determined. Variables with a VIP value > 0.8 were selected as most discriminant variables (Cox and Gaudard, 2013). Corresponding metabolites were identified with several databases: « in house » database, Human Metabolome Database (HMDB) (Wishart et al., 2007) and by the use of Chenomx Profiler software (Ellinger et al., 2013).

Statistical significance of identified discriminant metabolites was determined by integrating the ^1^H-NMR peaks of each metabolites contributing to the multivariate separation between groups. Integrals of each metabolite were normalized to spectral total area. The most appropriate statistical tests to compare variables between the different conditions were chosen to respect the two restrictive hypotheses allowing the use of the parametric tests: the normal distribution of the variable and the equality of the variances. Normality of data was determined for each group by a Shapiro-Wilk test, considering normality with an adjusted p-value > 0.05 (Shapiro and Wilk, 1965). Homoscedasticity of variances were determined by a Bartlett's test, considering homoscedasticity with a p-value > 0.05 (Bartlett, 1937). For normal and homoscedastic variables, statistical significance was determined using a one-way ANOVA. For non-normal or heteroscedastic variables, significance was determined using Dunn test. The significance was determined at p-value < 0.05 (*), p-value < 0.01 (**), and p-value <0.001(***). Heatmaps for both cellular extracts and extracellular fluids were constructed from mean values of discriminant metabolites integrals, using Excel functionalities (Microsoft^®^).

To highlight the most relevant altered pathways among all experimental conditions, an enrichment analysis was performed on the discriminant metabolites using the online available software MetaboAnalyst 4.0. for a metabolite set enrichment analysis (MSEA). (Chong et al., 2018). MSEA is a powerful tool used for metabonomic data interpretation, indicating which biological pathways are possibly linked to the identified metabolites. Thus, the MSEA describes the potential biological pathways related to the metabolic signature identified through our analysis. The suggested metabolic pathways are classified depending on the number of hit(s), corresponding to the number of imputed metabolites found into the identified pathways. Depending on the number of hits, an associated p-value is determined and can be under or above the 0.05 alpha value.

## Results

### Cell Viability

**Figure 1:**
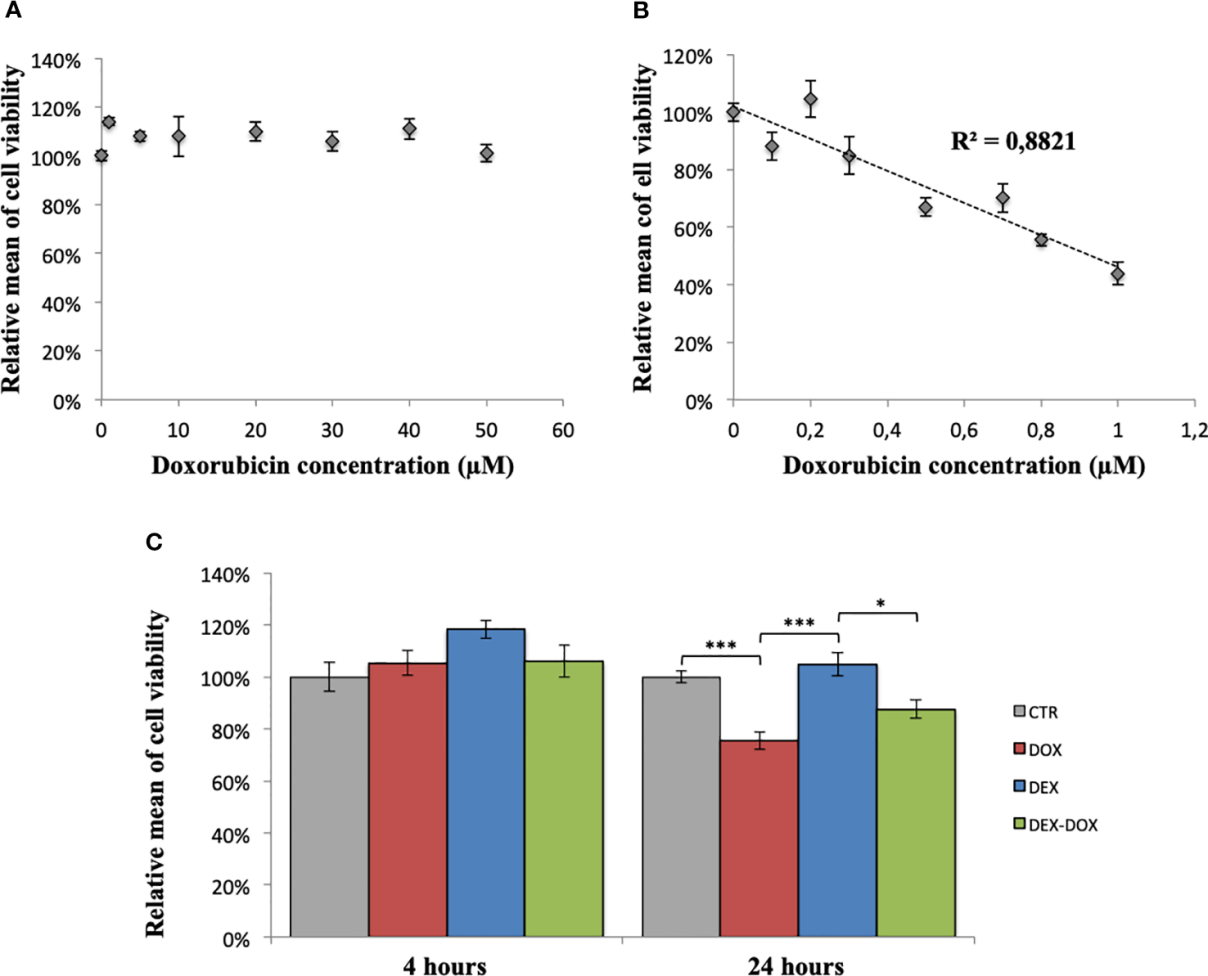
Relative mean of H9C2 cells viability measured by violet crystal assay. **(A)** After 4 hours of exposure to different doses of DOX. **(B)** After 24 hours of exposure to different doses of DOX. **(C)** Effect of DEX alone treatment and 30 minutes pre-incubation on both 4 and 24 hours of DOX exposure. One-way ANOVA with multiple comparisons: *p value < 0.05, ***p value < 0.001.

After 4 hours of exposure, DOX doses between 1 and 50 µM did not impact cell viability (**Figure 1A**). After 24 hours of exposure, DOX doses between 0,1 and 1 µM induced a dose-dependent decrease of cell viability (R^2^ = 0,88) (**Figure 1B**). The effect of a pre-incubation to DEX on cell viability was similarly assessed. After 4 hours of exposure, no significant change was observed between the four considered conditions (CTR, DOX 5 µM, DEX 50 µM, and DEX-DOX) (**Figure 1C**). After 24 hours of exposure, cells exposed to DOX were statistically different from controls. When cells were pre-incubated for 30 minutes with 3 µM DEX before DOX exposure, their viability was not statistically different from controls anymore (**Figure 1C**).

### Caspase 3 Activity

**Figure 2:**
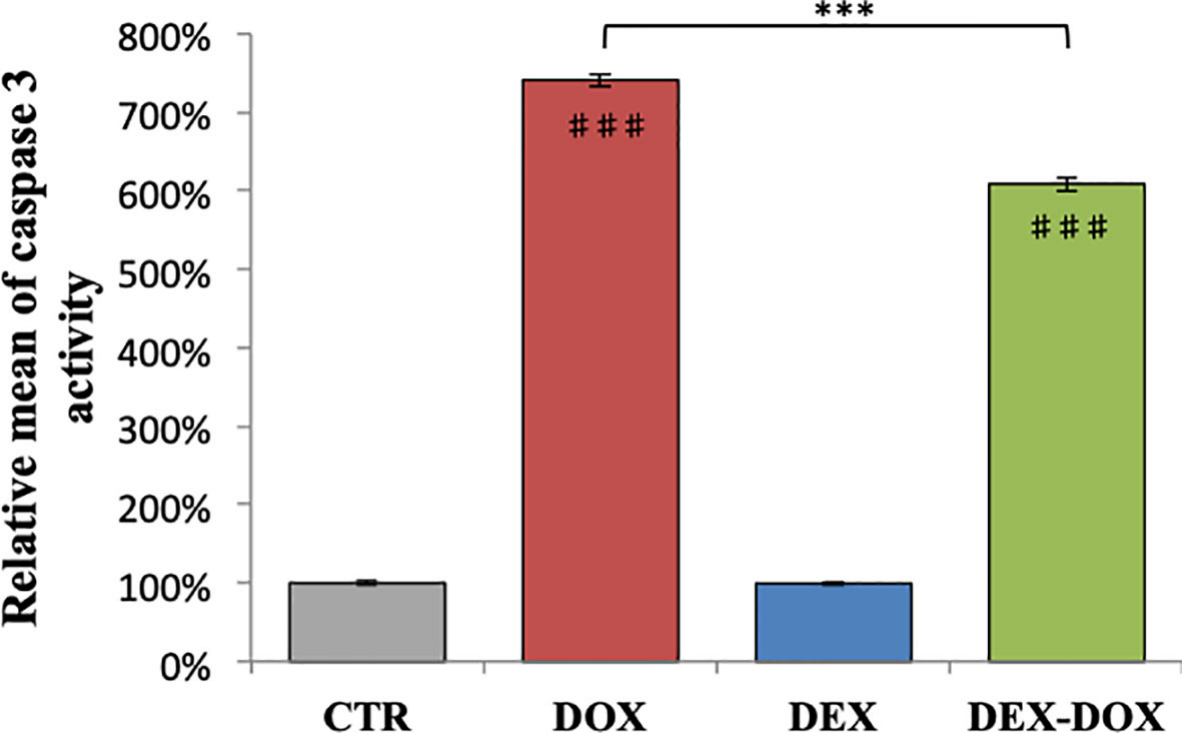
Relative mean of caspase 3 activity after 4 hours of DOX exposure. Effect of DEX alone treatment and 30 minute pre-incubation was also assessed. One-way ANOVA with multiple comparisons: ***p value < 0.001. ♯♯♯: comparison with CTR (p value < 0.001).

When H9C2 were exposed to 5 µM of DOX during 4 hours, the caspase 3 activity was statistically different from the control group and reached 700% of relative activity (**Figure 2**). When cells were pre-incubated for 30 minutes with 50 µM DEX before DOX exposure, the caspase 3 activity was statistically lower compared to the DOX group. However, the activity remained about 600% of controls.

### Oxidative Stress

**Figure 3:**
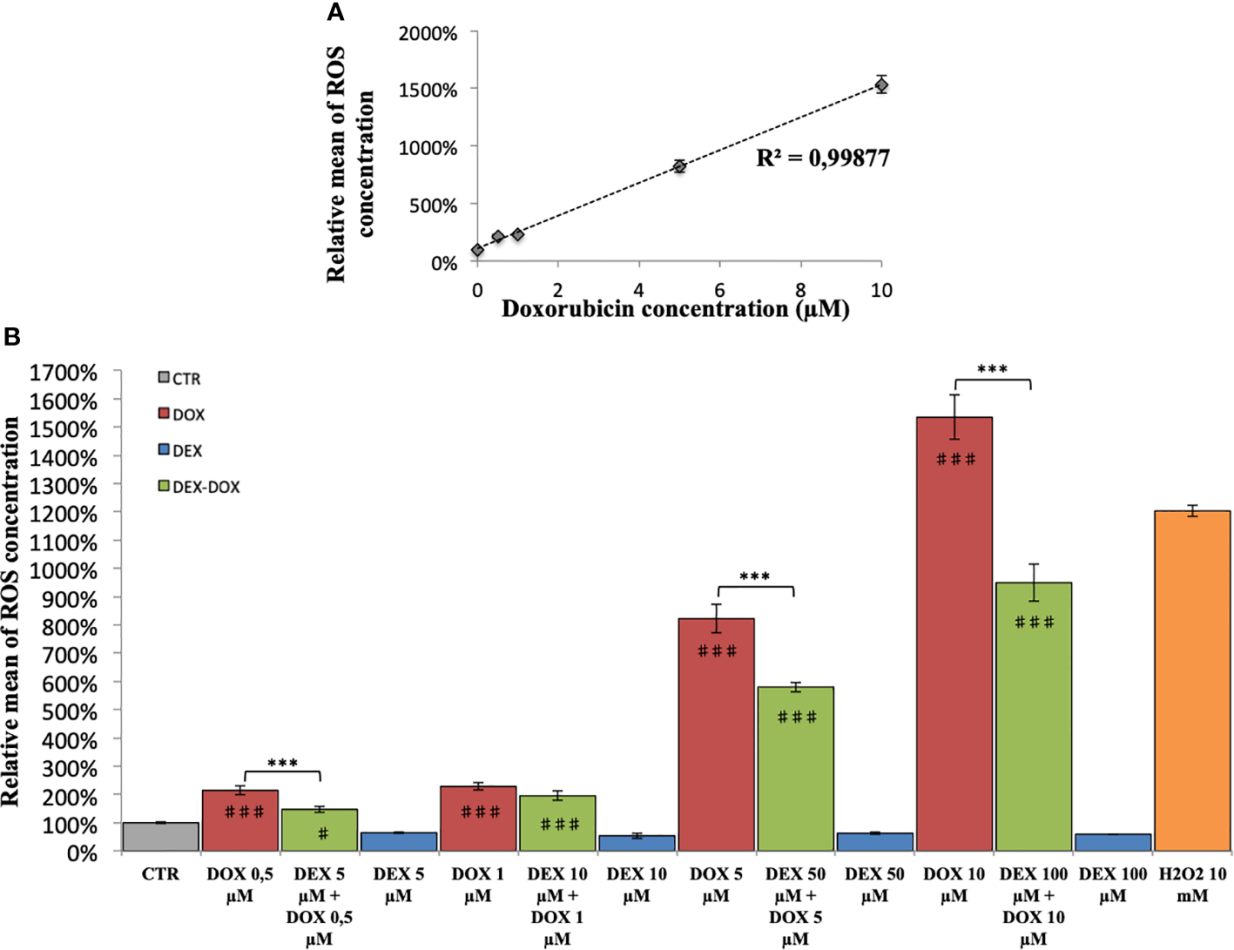
Relative mean of ROS concentration. **(A)** After 2 hours of exposure to different doses of DOX. **(B)** Effect of DEX alone treatment and 30 minute pre-incubation on 2 hours exposure to different doses of DOX. One-way ANOVA with multiple comparisons: ***p value < 0.001. ♯: comparison with CTR (p value < 0.05), ♯♯♯: comparison with CTR (p value < 0.001).

In H9C2 cells exposed to different DOX doses during 2 hours, the ROS production was proportional to DOX dose (R^2^ = 0.99) (**Figure 3A**). The effect of DEX on oxidative stress was also evaluated. Administered alone, DEX had no influence on ROS levels, compared to controls (**Figure 3B**). Administered 30 minutes prior to DOX exposure, there was a significant decrease in the amount of ROS produced as compared with cells exposed to DOX alone, for 3 of the 4 DEX-DOX dose pairs (10:1) tested (**Figure 3B**). However, all the DEX-DOX tested pairs remained statistically different from the CTR group, indicating that the ROS production did not recovered back to the basal production of the non-exposed cells.

### Metabonomic Study

**Figure 4:**
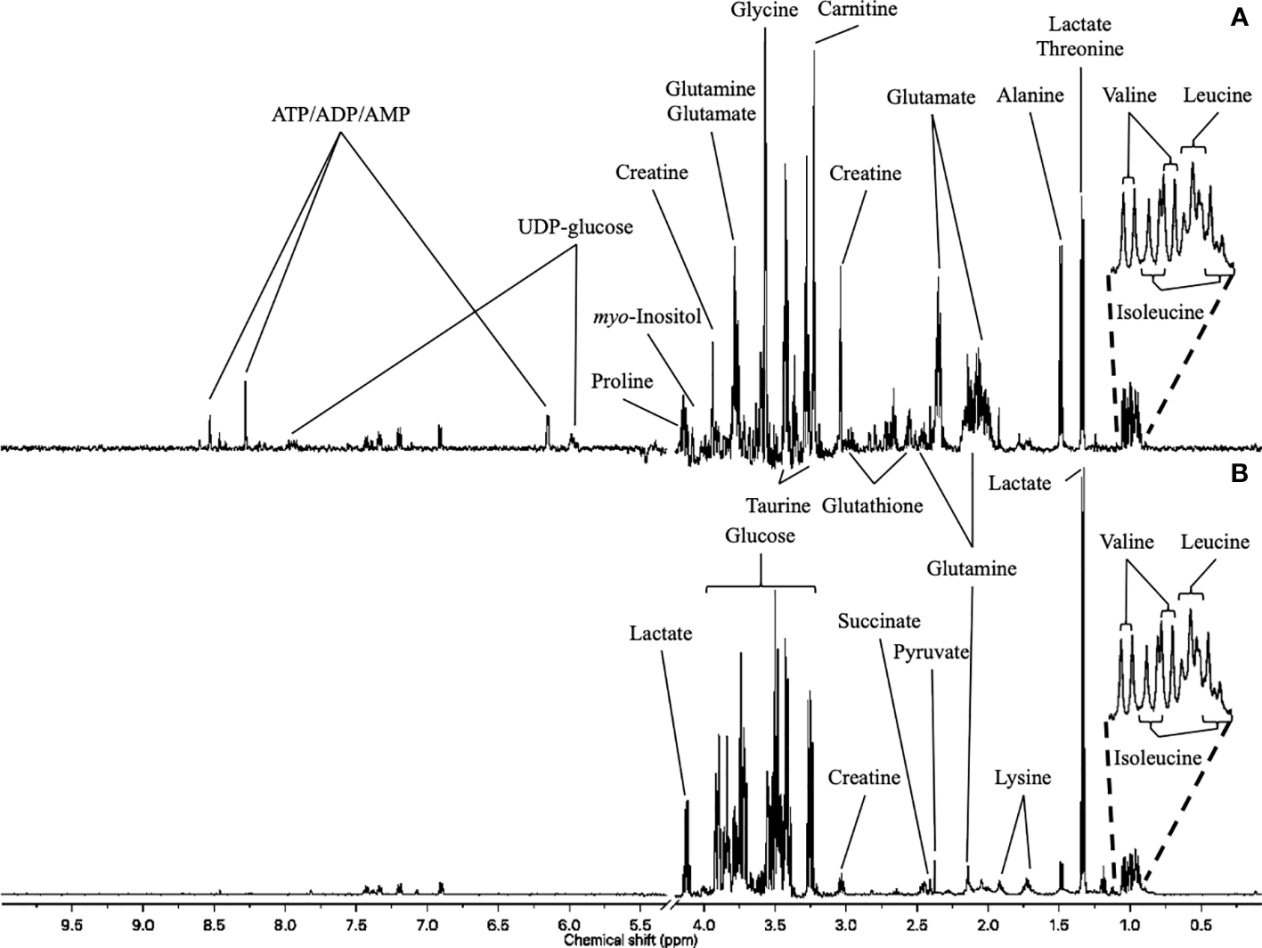
^1^H-NMR metabolome of H9C2 cells. **(A)** intracellular compartment (hydrophilic metabolites). **(B)** extracellular compartment (culture medium).

**Figure 5:**
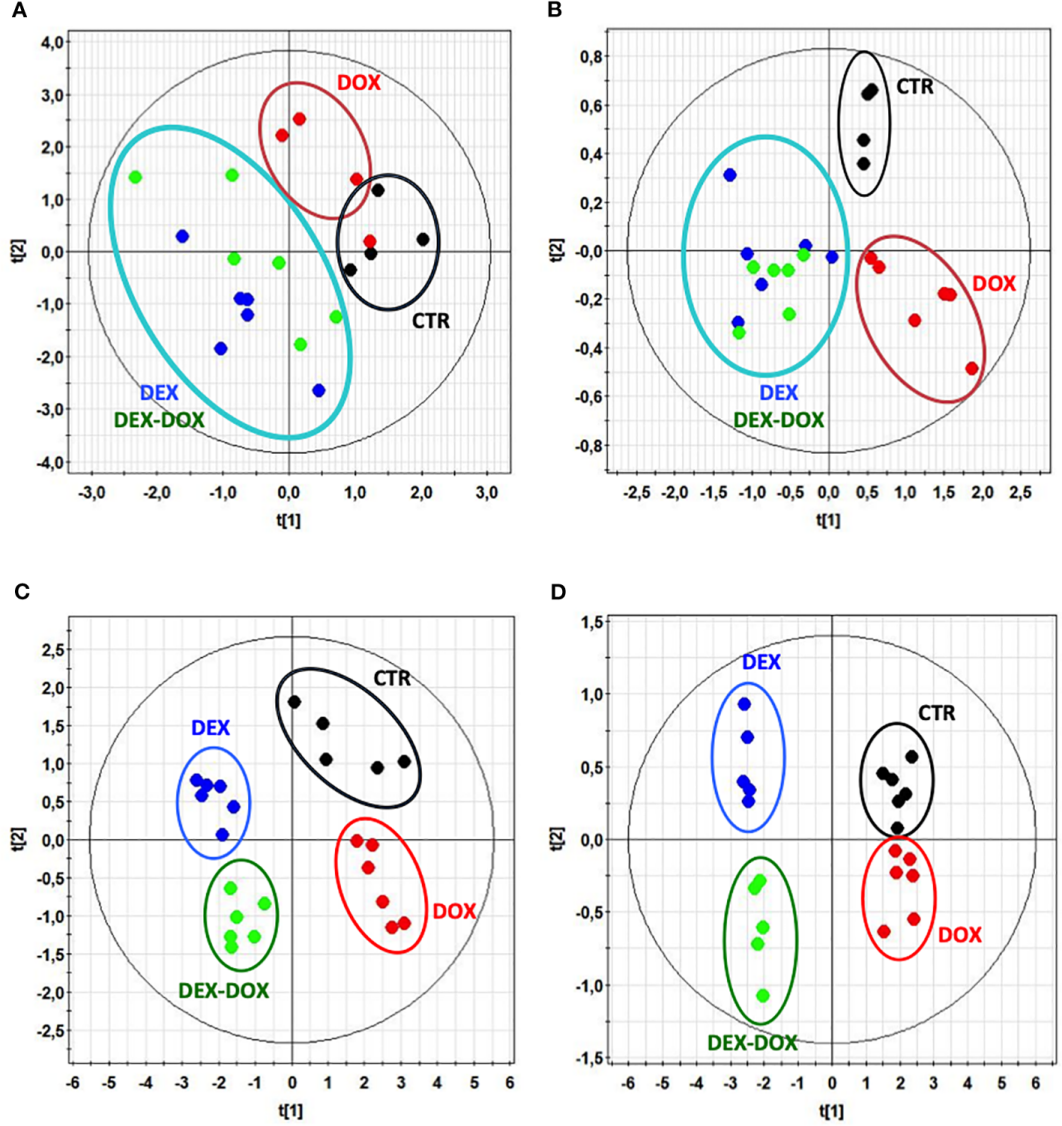
Scores plots of PLS-modeled metabonomic data: control group (•) vs DOX group (•) vs DEX group (•) vs DEX-DOX group (•). **(A)** Intracellular extracts of 4 hours exposure. R^2^_cum_ = 0.36; Q^2^_cum_ = 0.04; Hotteling's T2 = 0.95; p-value (CV-ANOVA) > 0.05. **(B)** Culture media of 4 hours exposure. R^2^_cum_ = 0.69; Q^2^_cum_ = 0.45; Hotteling's T2 = 0.95; p-value (CV-ANOVA) < 0.05. **(C)** Intracellular extracts of 24 hours exposure. R^2^_cum_ = 0.59; Q^2^_cum_ = 0.54; Hotteling's T2 = 0.95; p-value (CV-ANOVA) < 0.05. **(D)** Culture media of 24 hours exposure. R^2^_cum_ = 0.59; Q^2^_cum_ = 0.52; Hotteling's T2 = 0.95; p-value (CV-ANOVA) < 0.05.

^1^H-NMR metabolic signatures of DOX exposure and DEX pre-incubation on H9C2 cells were obtained for two exposure times, using PLS-DA multivariate data analysis on both intra- and extra-cellular fluids spectra (**Figure 4**). For the 4 hours exposure, the scores plots show a separation among the experimental conditions. However, no discrimination of DEX group from DEX-DOX group can be seen, suggesting that these two groups have a similar metabolic profile clearly different from DOX and CTR groups (**Figures 5A, B**). The scores plots of both extra- and intra-cellular contents indicate a clear separation among the four conditions for the 24 hours exposure, suggesting four different metabolic profiles (**Figures 5C, D**).

To specify the most relevant discriminant metabolites, several PLS-DA models were constructed to compare DOX vs CTR groups and DEX-DOX vs DOX groups to highlight the major metabolic changes induced by DOX exposure and effects of DEX pre-incubation (**Table 1**). From these comparisons, variables (chemical shifts) with a VIP score > 0.8 were considered as the most discriminant ones and the corresponding metabolites were identified (**Tables 2** and **3**).

**Table 1:** Parameters of carried out PLS-DA modeled comparisons.

Time of exposure	Matrix	Compared groups	Models parameters		
			R^2^_cum_	Q^2^_cum_	P value (CV-ANOVA)
**4 hours**	Aqueous phase of intracellular extracts	CTR vs DOX	0.9	0.45	>0.05
	Aqueous phase of intracellular extracts	DEX-DOX vs DOX	0.81	0.53	>0.05
	Extracellular fluid	CTR vs DOX	0.88	0.74	>0.05
	Extracellular fluid	DEX-DOX vs DOX	0.98	0.89	<0.05
**24 hours**	Aqueous phase of intracellular extracts	CTR vs DOX	0.95	0.84	<0.05
	Aqueous phase of intracellular extracts	DEX-DOX vs DOX	0.99	0.97	<0.05
	Extracellular fluid	CTR vs DOX	0.88	0.8	<0.05
	Extracellular fluid	DEX-DOX vs DOX	0.98	0.97	<0.05

**Table 2:** Identified discriminant metabolites with corresponding chemical shifts for the 4 hour exposure.

Metabolites	Chemical shifts (ppm)	DOX (compared to CTR)	DEX-DOX (compared to DOX)
**1. AQUEOUS PHASES OF INTRACELLULAR EXTRACTS:**			
AMP/ADP/ATP	6.15 (t) 8.28 (s) 8.60 (s)	↓ (0.89)	–
Alanine	1.48 (d	–	↓ (0.89)
Carnitine	3.22 (s) 3.40 (s)	↓ (2.47)	↓ (2.93)
Citrate	2.53 (d) 2.67 (d)	↓ (0.97)	↑ (1.42)
Creatine	3.05 (s) 3.94 (s)	↑ (1.19)	↓ (0.87)
Glutamate	2.10 (m) 2.36 (m) 3.77 (m)	↓ (2.86)	↓ (3.46)
Glutamine	2.12 (m) 2.45 (m) 3.76 (m)	↑ (1.73)	↓ (5.01) *
Glutathion	2.15 (m) 2.55 (m) 3.00 (t)	↓ (1.21)	–
Glycerophosphocholine	3.24 (s)	↓ (3.83) **	↑ (2.06) *
Glycine	3.57 (s)	↑ (2.07)	↑ (2.50)
Guanidoacetate	3.80 (s)	↓ (0.85)	↓ (5.10)
Isoleucine	0.94 (t) 1.04 (d) 1.26 (m) 1.45 (m) 3.65 (d)	↓ (2.90)	↓ (1.16)
Lactate	1.32 (d) 4.12 (q)	↓ (1.33)	↑ (1.60)
Leucine	0.94 (t) 1.70 (m) 3.72 (m)	↓ (1.89)	–
*Myo*-inositol	3.52 (m) 3.60 (m) 4.07 (t)	↑ (3.25)	↓ (2.36)
Phosphocholine	3.23 (s)	–	↓ (2.06) *
Phosphocreatine	3.06 (s) 3.94 (s)	↓ (1.19)	↑ (0.87)
Proline	2.01 (m) 2.35 (m) 3.33 (m) 4.10 (m)	↑ (2.56)	↑ (1.27)
Serine	3.95 (m) 4.00 (m)	↑ (2.03)	–
Taurine	3.25 (t) 3.43 (t)	↓ (3.83) *	↑ (2.93)
Threonine	1.32 (d) 4.25 (m)	↑ (1.33)	↓ (1.60)
UDP-glucose	3.50 (m) 3.80 (m) 4.20 (m) 5.98 (t) 7.95 (d)	↑ (2.17)	↑ (3.62)
**2. EXTRACELLULAR FLUIDS:**			
Alanine	1.48 (s)	↑ (1.13)	–
Creatine	3.05 (s)	↓ (0.93) *	–
Glucose	3.23 (dd) 3.39 (m) 3.45 (m) 3.52 (dd) 3.72 (m) 3.82 (m) 3.88 (dd) 5.22 (d)	↓ (4.58)	↑ (6.57) *
Glutamine	2.12 (m) 2.44 (m) 3.76 (t)	↑ (1.31)	↑ (1.25) *
Isoleucine	0.94 (t) 1.04 (d) 1.26 (m) 1.45 (m) 3.65 (d)	↑ (1.34)	↓ (1.68) *
Lactate	1.32 (d) 4.12 (q)	↓ (1.88)*	↑ (1.19)
Lysine	1.70 (m) 1.90 (m)	↑ (1.18)	–
Phosphocreatine	3.06 (s) 3.94 (s)	↓ (0.93) *	–
Proline	2.01 (m) 2.35 (m) 3.33 (m) 4.10 (m)	↑ (1.09)	–
Pyruvate	2.38 (s)	–	↑ (2.26) **
Succinate	2.40 (s)	↓ (0.84)	↑ (3.09)
Valine	0.99 (dd) 2.25 (m) 3.60 (d)	↑ (1.56)	↓ (1.39) *

Peaks multiplicity is indicated between brackets (s, singulet; d, doublet; t, triplet; m, multiplet). Metabolites concentration changes are indicated by arrows. VIP values are precised between brackets. Dunn test: *p value < 0.05, **p value < 0.01.

**Table 3:** Identified discriminant metabolites with corresponding chemical shifts for the 24 hours exposure.

Metabolites	Chemical shifts (ppm)	DOX (compared to CTR)	DEX-DOX (compared to DOX)
**1. AQUEOUS PHASES OF INTRACELLULAR EXTRACTS:**			
Alanine	1.48 (d)	–	↓ (1.67)
Glutamate	2.10 (m) 2.36 (m) 3.77 (m)	–	↑ (1.38)
Glutamine	2.12 (m) 2.45 (m) 3.76 (m)	↑ (2.69)**	↓ (1.72)
Glycerophosphocholine	3.24 (s)	–	↑ (1.32)***
Glycine	3.57 (s)	–	↑ (2.17)*
Isoleucine	0.94 (t) 1.04 (d) 1.26 (m) 1.45 (m) 3.65 (d)	↑ (1.09)	–
Lactate	1.32 (d) 4.12 (q)	↑ (5.38)*	↓ (5.63)***
Leucine	0.94 (t) 1.70 (m) 3.72 (m)	↑ (1.09)	↑ (1.02)
Methionine	2.14 (s) 2.65 (t) 3.85 (t)	↑ (5.06)	↓ (1.58)
Phosphocholine	3.23 (s)	–	↑ (1.32)***
Phosphocreatine	3.05 (s)	↑ (0.93)*	↑ (1.21)***
Proline	2.01 (m) 2.35 (m) 3.33 (m) 4.10 (m)	↑ (1.13)	↓ (1.61)
Serine	3.95 (m) 4.00 (m)	–	↑ (8.79)***
Taurine	3.25 (t) 3.43 (t)	↑ (5.38)***	↓ (4.90)***
UDP-glucose	3.50 (m) 3.80 (m) 4.20 (m) 5.98 (t) 7.95 (d)	↓ (0.88)	↑ (1.44)
**2. EXTRACELLULAR FLUIDS:**			
Glucose	3.23 (dd) 3.39 (m) 3.45 (m) 3.52 (dd) 3.72 (m) 3.82 (m) 3.88 (dd) 5.22 (d)	↓ (6.62)	↓ (2.68)
Glutamine	2.12 (m) 2.44 (m) 3.76 (t)	↑ (2.49) ***	↓ (0.13)*
Isoleucine	0.94 (t) 1.04 (d) 1.26 (m) 1.45 (m) 3.65 (d)	↑ (1.07)	↑ (1.24)
Lactate	1.32 (d) 4.12 (q)	↑ (7.03)	↓ (12.00)***
Pyruvate	2.38 (s)	↓ (0.80)	–
Serine	3.83 (dd) 3.96 (m)	–	↑ (2.71)***
Succinate	2.40 (s)	↑ (0.81)	↑ (2.80)*
Valine	0.99 (dd) 2.25 (m) 3.60 (d)	↑ (1.27)	–

Peaks multiplicity is indicated between brackets (s, singulet; d, doublet; t, triplet; m, multiplet). Metabolites concentration changes are indicated by arrows. VIP values are precised between brackets. Dunn test: *p value < 0.05, **p value < 0.01, ***p value < 0.001.

**Figure 6:**
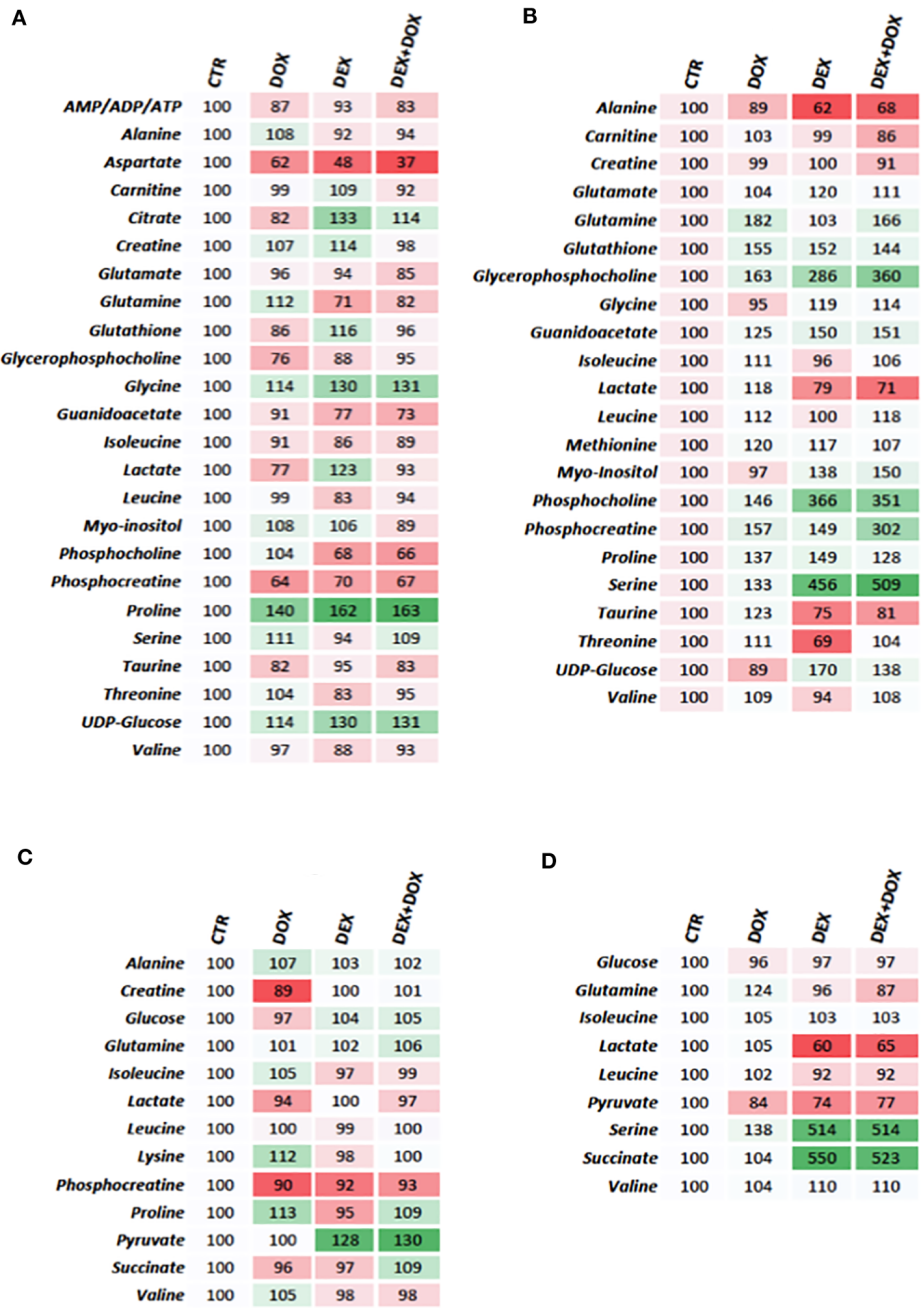
Heatmaps constructed from relative means of discriminant metabolites normalized integrals. **(A)** H9C2 intracellular compartment after 4 hours of DOX/DEX exposure. **(B)** H9C2 intracellular compartment after 24 hours of DOX/DEX exposure. **(C)** H9C2 culture media after 4 hours of DOX/DEX exposure. **(D)** H9C2 culture media after 24 hours of DOX/DEX exposure.

Heatmaps were constructed using normalized AUC from identified discriminant metabolites, for both extra- and intra-cellular compartments. In each case, the value is the relative mean of metabolite AUC (**Figure 6**).

**Figure 7:**
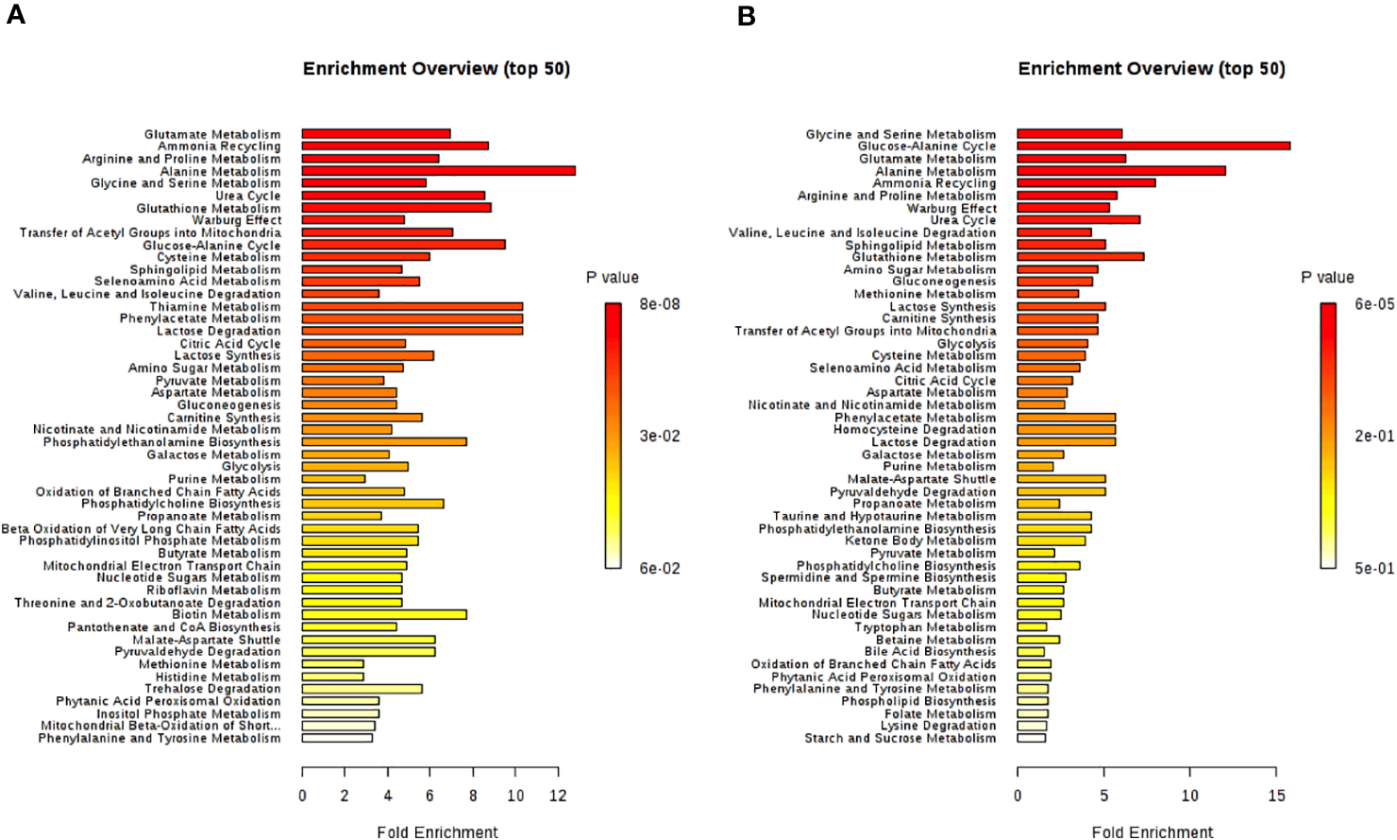
Metabolite set enrichment analysis performed on ^1^H-NMR metabonomic data, using MetaboAnalyst 4.0 online software. **(A)** 4 hours H9C2 cells exposure to DOX/DEX. **(B)** 24 hours H9C2 cells exposure to DOX/DEX.

All identified discriminant metabolites were then imputed into the MetaboAnalyst 4.0 online software for a MSEA. This analysis highlighted the most likely metabolic pathways suggested by ^1^H-NMR metabonomic data (**Figure 7**). Thereby, glucose metabolism and different amino acid metabolisms (alanine, glutamate, glycine, serine, proline, arginine, valine, leucine, and isoleucine) are strongly involved in the metabonomic signatures of H9C2 cells exposed to DOX or/and DEX. The enrichment analysis also pointed out glutathione, phenylacetate, and thiamine metabolisms as well as lactate degradation.

## Discussion

In this study, for a short time of exposure (4 hours), DOX did not jeopardize H9C2 cell viability. For longer period of exposure (24 hours) however, DOX caused a dose-dependent decrease of cell viability, at doses less than 1 µM. Interestingly, under the same experimental conditions, pre-exposing the cells to DEX did improve cell viability.

Our results also showed that DOX can trigger the apoptotic process in H9C2 cells, as suggested by the significant activation of caspase 3 enzyme, an effect already reported by others (Porter and Jänicke, 1999). The protective effect of DEX already noticed when assessing cell viability was confirmed by its ability to counteract the DOX-induced apoptotic effect in H9C2 cells. Similarly, the higher levels of ROS detected in H9C2 cells exposed to DOX, were largely prevented when cells were pre-incubated with DEX, suggesting an attenuation of oxidative stress in H9C2 cells.

H9C2 cell line is a rat cardiomyoblast-derived cell line that has some similarities with differentiated cardiomyocytes but does not have cardiac contractile and functional characteristics. Therefore this cell line is not the most extrapolable *in vitro* model for studying human DOX-induced cardiac metabolism alterations and protective effects of dexrazoxane. However, this cellular model was used in this study due to the constraints imposed by the ^1^H-NMR metabonomic approach. Indeed, ^1^H-NMR spectroscopy suffers from a poor sensitivity, requiring more than 10 million cells per analysis to obtain a correct signal/noise ratio in an acceptable acquisition time. H9C2 cells allowed to satisfy those technical constraints. For this purpose, we ensured, thanks to our own experimental data and through a thorough literature review, that H9C2 cells is a model able to reproduce the main features of the toxic mode of action of DOX. Overall, all the cytotoxic indicators assessed in this study were positive in DOX-exposed H9C2 cells. Our results are in agreement with previous reports on the mode of toxic action of DOX incriminating the triggering of oxidative stress leading to cell apoptosis, among other effects. Indeed, Cheung et al. (2015) and Shabalala et al. (2019) previously demonstrated the DOX-induced oxidative stress in H9C2 cells (Cheung et al., 2015; Shabalala et al., 2019). Li et al. (2013) and Moreira et al. (2014) highlighted apoptosis in H9C2 cells exposed to DOX (Li et al., 2013; Moreira et al., 2014). Topoisomerases inhibition and sarcoplasmic reticulum stress were also observed in H9C2 cells exposed to DOX by other authors (Lyu et al., 2007; Lou et al., 2015). Moreover, our data support the beneficial effects of DEX on the oxidative stress and, consequently, on apoptosis, as previously established by several authors. All together, those results suggest, despite some obvious drawbacks, that the H9C2 cell line is a relevant model for studying DOX cardiotoxicity and the protective effects of DEX. Indeed, H9C2 cells are able to mimic the important well-known mechanisms of DOX cardiotoxicity: oxidative stress, apoptosis, cell death, topoisomerases inhibition, and sarcoplasmic reticulum stress. Hence, because a metabonomic study requires a very high amount of cells, we decided to investigate metabolic changes caused by DOX and DEX in this cardiomyoblast *in vitro* model characterized by its high proliferative rate.

Metabolic effects of DOX and pre-incubation with DEX were observed at two different incubation times, 4 and 24 hours, using a ^1^H-NMR based metabonomic approach. Both intra- and extra-cellular compartments of H9C2 cells were considered for this purpose. Exposure doses were selected based on the cell viability assays discussed above. An *in vitro* metabonomic study requires an important number of living cells to ensure a adequate signal-to-noise ratio in the NMR spectra. Accordingly, the DOX doses and exposure periods were carefully selected not to exceed 20% of cell death. As for the selection of DEX doses, clinical practices were reproduced with a DEX/DOX ratio of 10, with a pre-incubation time of 30 minutes before the addition of DOX (Langer, 2014).

The metabonomic profiles obtained under such conditions pointed out a global alteration of the energy metabolism, as suggested by the decreased intracellular phosphocreatine (PCr) level and the increased creatine (Cr). PCr is produced by phosphorylation of Cr, using ATP, and plays the role of local energy storage for muscle contraction (Wyss and Kaddurah-Daouk, 2000). ATP provides the energy needed to supply many biochemical reactions. Its intracellular stock needs to be regularly renewed to sustain cell physiological activity (Murray and Domenjoud, 2008). A decrease of PCr may suggest a lower ATP production or a higher ATP consumption.

The impairment in ATP production revealed by the metabonomic assessment seems to involve several steps. First, a defect in the glycolytic activity is indicated by lower intra- and extra-cellular levels of end product of glycolysis (Rogatzki et al., 2015). Niu et al. also reported a decrease in end product of glycolysis by an ^1^H-NMR metabonomic investigation of urine, *serum,* and heart extracts from male Sprague Dawley rats exposed to DOX (Niu et al., 2016). However, in Wistar rats exposed to an acute DOX dose, no change in lactate and glucose levels were found in myocardial extracts (Andreadou et al., 2009). Next, the increase of uridine diphosphate glucose (UDP-glucose), byproduct of glucose for glycogenogenesis (Adeva-Andany et al., 2016), attests the use of glucose for non-glycolytic pathways. Also, a Krebs cycle dysfunction is suggested by the decreased levels of two cycle intermediates (Akram, 2014): citrate in the intracellular compartment and succinate in extracellular compartment. Moreover, other metabolic changes are indirect witnesses of citric acid cycle deficiency: the enhancement of pyruvate precursors and byproducts levels (alanine, glycine, serine, and threonine) implying a non-consumption of pyruvate by the mitochondria; the increase of α-ketoglutarate precursors (glutamine and proline) levels both in the intracellular and extracellular compartments; the extracellular accumulation of leucine, isoleucine, and valine, essential amino acids less consumed for Krebs cycle supplying. Decreases in leucine, isoleucine, and valine levels in myocardial extracts, similar to our observations in the intracellular compartment, were also reported in an *in vivo* study using male Wistar rats exposed to DOX (Andreadou et al., 2009). Finally, metabonomic results also indicate an alteration of β-oxidation by the decreased intracellular content of carnitine and a lower consumption of its precursor (lysine). A similar metabolic alteration characterized by a reduction in carnitine level in heart extracts and an enhanced *serum* level of lysine was also observed in Sprague Dawley rats exposed to DOX (Niu et al., 2016).

After 24 hours of exposure to DOX, a metabolic switch for energy production was evidenced by higher levels of lactate in both the intra- and extra-cellular compartments and this effect was associated with a higher consumption of glucose from culture medium, a lower alanine production and a decreased pyruvate secretion in culture medium, suggesting a glycolytic stimulation with a lactate dehydrogenase activation for redox coenzymes regeneration. The reduced levels of glucose-derived metabolites, such as UDP-glucose and *myo*-inositol, in the intracellular compartment confirm the glycolytic use of glucose. The changes observed in glutamine and essential amino acids such as leucine, isoleucine, and valine also support the general depression in mitochondrial energy metabolism. The enhancement of lactate level was also highlighted by Kawasaki et al. in Sprague Dawley rats exposed to DOX, 24 hours after the last DOX injection. However this enhancement was accompanied by an increased level of alanine which was not found in our study (Kawasaki et al., 1996). This alteration of the mitochondrial metabolism was also reported by several authors in animal and other *in vitro* models. Carvalho et al. highlighted an inhibition of mitochondrial metabolism associated with a stimulation of glycolysis in male Wistar Hans rats exposed subchronically to DOX (Carvalho et al., 2010). For this purpose, these authors monitored the metabolization of ^13^C-labeled glycolytic precursors and products in perfused rat hearts, using ^13^C-NMR. Niu et al. mentioned an alteration in fatty acids metabolism and mitochondrial dysfunction by an ^1^H-NMR metabonomic investigation of urine, *serum,* and hearth extracts from male Sprague Dawley rats exposed to DOX (Niu et al., 2016). Chaudhari et al. described an accumulation of acetate and pyruvate in extracellular fluids of human induced pluripotent stem cell-derived cardiomyocytes (hiPSC-CM) analyzed by ^1^H-NMR spectroscopy, also supporting a dysfunction of the mitochondrial metabolism with lower ATP production (Chaudhari et al., 2017). Energy metabolism is closely linked to cardiac function and there are evidences that a pathological event occurring in heart leads to an abnormal energy metabolism, especially consecutively to a mitochondrial impairment. A change in metabolites pattern involved in energy metabolism is suggested as an early cardiotoxicity hallmark and could therefore be used to identify predictive biomarkers (Li et al., 2015).

DEX pre-incubation was able to largely counteract the DOX effects on cell energy metabolism, as indicated by the increase of PCr intracellular level, reflecting a higher production of ATP thanks to a better citric cycle activity. This effect was proved by the enhancement of intracellular citrate content, the higher succinate secretion and the bigger consumption of glutamine and essential amino acids such as leucine, isoleucine, and valine. Moreover, the metabonomic observations indicated that under our experimental conditions the citric cycle used more non-glytolytic substrates as amino and fatty acids than glucose. Indeed, the higher extracellular level of glucose suggests a lower consumption by the cells while the ATP production is more stimulated. A similar observation was done when H9C2 cells were pre-exposed to DEX before a 24 hours exposure to DOX. The metabolic changes indicate a stimulation of Krebs cycle with use of non-glycolytic substrates. Carvalho et al. also observed a protective effect of DEX by counteracting the depletion of mitochondrial metabolism induced by a repeated DOX exposure in male Wister Hans rats (Carvalho et al., 2010).

Under DOX exposure, the triggering of an adaptive metabolism against oxidative stress was also revealed, mainly from the stimulation of the biosynthesis of glutathione (GSH) and its elevated use as an antioxidant by the cells. Indeed, GSH intracellular content is decreased, suggesting that GSH is highly used to convert hydrogene peroxide into water, by the glutathione peroxidase (Lubos et al., 2011). This is a cell response to oxidative stress induced by DOX, as demonstrated by the *in vitro* ROS production. The intracellular changes observed in glutamate, glycine and glycine-related metabolites (taurine and guanidoacetate) indicate an induced GSH biosynthesis by the cells. Glutathione is a tripeptide, composed of glutamate, glycine, and cystein. It is found in all mammal tissues and is a powerful agent for redox homeostasis maintenance (Lu, 2013). Moreover, the decreased intracellular content of carnitine may be associated with its use as an antioxidant (Gülçin, 2006), such as the decreased of intracellular taurine concentration (Schaffer et al., 2010).

After 24 hours of exposure to DOX, the synthesis of GHS was still stimulated, as suggested by the intracellular changes of its precursors (glutamate and glycine) contents and its increased intracellular concentration. However, GSH was more concentrated in DOX exposed cells than controls, indicating that the oxidative stress is fading, compared to the 4 hour timeframe. The increased levels of taurine and carnitine may also suggest a higher synthesis of these antioxidant compounds for redox state maintenance and a lower consumption because of the fading oxidative stress.

Metabonomic data also highlighted the antioxidant potential of DEX, as demonstrated by the decreased intracellular GSH content, reflecting its lower consumption as a redox maintenance agent. Lower consumptions of glutamate and glycine, both GSH precursors, were also observed, indicating a decreased GSH synthesis. Moreover, an enhanced concentration of taurine may be due to a decreased oxidative stress. These metabolic changes are in adequacy with the ROS quantification showing a reduced oxidative stress when cells were pre-exposed to DEX.

In parallel to the shift in energy production, the increase of intracellular phosphocholine and glucose-derived *myo*-inositol, precursors of phosphatidylcholine and phosphatidylinositol phospholipids, suggested an enhancement of phospholipids biosynthesis in H9C2 cells exposed to DOX. These metabolic changes most likely sustain the needs for the higher turnover of biological membranes in response to DOX-induced oxidative stress. Indeed, membrane phospholipids are a major target of peroxidation by free radicals, leading to a loss of membrane integrity (Burton and Jauniaux, 2011). Therefore, enhancing phospholipids biosynthesis may be seen as an adaptive metabolic change to counteract oxidative stress. A change in phospholipids metabolism is reported as an important metabolic change occurring when cardiotoxic drugs are damaging the integrity of cell membranes. Modifications in phospholipids-associated metabolites pattern is suggested to be considered for early biomarkers identification (Li et al., 2015).

After 24 hours of DOX exposure, the increase of intracellular phosphocholine and glycerophosphocholine levels indicate an ongoing activation of the Kennedy's pathway, which is responsible of phophatidylcholine synthesis, a major phospholipid found in biological membranes (Gibellini and Smith, 2010). Again, this metabolic activation can be considered as an adaptive change to counteract a dwindling oxidative stress. Metabolic changes in choline, phosphocholine, and *myo*-inositol were also found in heart extracts and *serum* of Sprague Dawley rats exposed to DOX. The authors mentioned a reduced level of choline in heart extracts and an enhanced *myo*-inositol quantity in *serum*, suggesting their consumption for enhancing phospholipids synthesis (Niu et al., 2016).

In case of pre-exposure to DEX, the metabolic changes describe a reduced phospholipids synthesis in the 4 hour exposure protocol. Indeed, the decreased intracellular concentration of phosphocholine associated with an increased concentration of its precursor, glycine, as well as a higher glycerophosphocholine content support the hypothesis of a reduced synthesis of phosphatidylcholine. Moreover, less intracellular myo-inositol associated with a lower glucose consumption indicate a reduced synthesis of phosphatidylinositol. However, the data obtained for a longer period of exposure highlighted an important DEX-induced stimulation of phospholipids synthesis.

Methionine is an essential amino acid which is very important for protein synthesis. Indeed, this compound corresponding to the « start » codon of a mRNA is always incorporated at the first position during its translation. In H9C2 cells, DOX caused increase of intracellular level of methionine, suggesting an altered synthesis of proteins or an enhancement of proteins catabolism.

**Figure 8:**
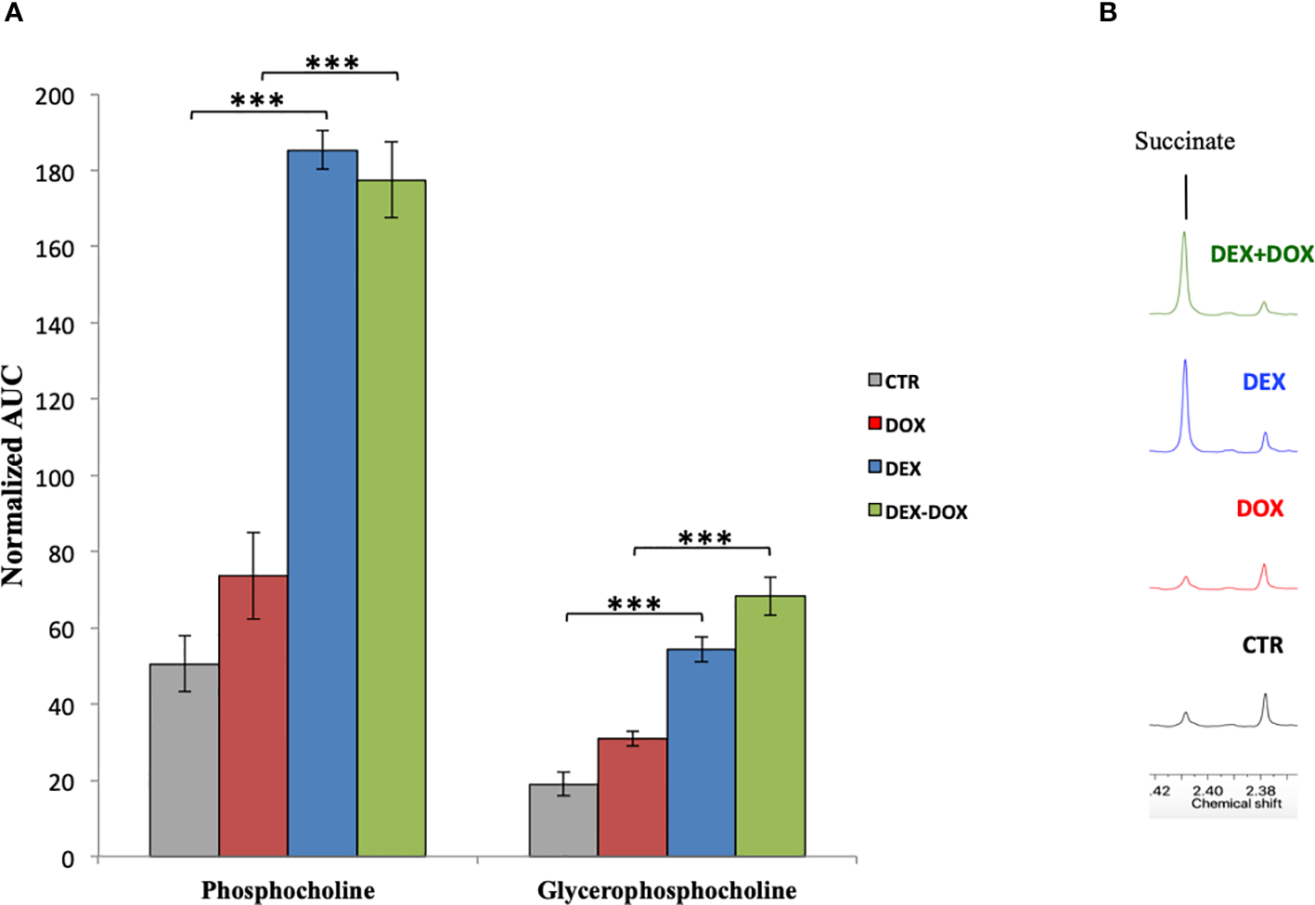
**(A)** Mean normalized AUC of phosphocholine and glycerophosphocholine peaks in the ^1^H-NMR spectra of intracellular compartment. **(B)** Enlargement of culture media ^1^H-NMR spectra showing the over-secretion of succinate. ***p value < 0.001.

As already discussed, DEX is able to some extent to improve cell viability against DOX. The metabonomic evaluation also showed metabolic changes possibly in relation with cell survival, growth, and proliferation. First, DEX caused a stimulation of Kreb's cycle and ATP production associated with an important synthesis of membrane phospholipids. Given that the ROS quantification demonstrated a clear antioxidant effect of DEX, this important phospholipids biosynthesis cannot be connected with a compensatory repair mechanism of peroxidized membranes. Secondly, the Kennedy's pathway (phosphatidylcholine synthesis) is highly activated by DEX exposure (**Figure 8A**). The choline kinase, first enzyme of this pathway, can be activated by the signaling pathway RAS/PI3K which promotes cell survival and proliferation (Gibellini and Smith, 2010). RAS is able to activate the PI3K which converts PIP2 to PIP3. PIP2 is a phosphatidylinositol-derived compound. Given that the metabonomic results highlighted a stimulation of phosphatidylinositol synthesis necessary for this purpose, the involvement of RAS/PI3K pathway is highly suspected as a possible cardioprotective mechanism of DEX. PIP3 synthesized by PI3K is able to activate the AkT protein which in turn activates the mTOR transcription factor. mTOR is an important regulator of protein synthesis, cell cycle, and cytoskeleton development. An over-activation of PI3K/AkT/mTOR pathway is responsible of a cell proliferation stimulation (Dreyer et al., 2009).

Moreover, the metabolic analysis of the culture media highlighted an important secretion of succinate in cells pre-exposed to DEX (**Figure 8B**). In addition to its involvement in the energy metabolism, succinate could act as a communication molecule *via* its membrane receptor GPR91, a G-protein coupled receptor capable of activating the PI3K/AkT/mTOR pathway (Yang et al., 2016). Recent studies reported that succinate is capable of binding and activating the GPR91 membrane receptor. This receptor is expressed in kidney, liver, heart, and possibly other tissues (de Castro Fonseca et al., 2016). Succinate is involved in major functions such as blood pressure regulation and lipolysis inhibition. Some authors demonstrated that succinate causes cardiomyocyte hypertrophy during a heart ischemic event (Aguiar et al., 2014). GPR91 seems to be implicated in hypertrophic cardiac diseases and thus could be a potential target to counteract heart diseases implying a loss in cardiomyocytes population, such as heart infarct and DOX-induced cardiotoxicity. Moreover, GPR91 expression in H9C2 cells has been recently demonstrated (Li et al., 2017; Lu et al., 2018). Therefore, we anticipate that this receptor could be highly implicated in DEX cardioprotective mechanisms. Studying its role as a cardiac cells survival inducer and, as such, a possible future possible target to develop new cardioprotective agents will be our next challenges.

## Conclusion

First, the H9C2 cell line was experimentally validated as an adequate *in vitro* model to assess DOX toxic mode af action, including oxidative stress and apoptosis. Then, the cardioprotective potential of DEX was confined in this *in vitro* model. Finally, the ^1^H-NMR-based metabonomics assessment highlighted several metabolic pathways involved in DOX cardiotoxicity and DEX cardioprotective effects as potential metabolic targets for cardioprotection: energy metabolism, redox maintenance, phospholipids, and proteins metabolism. Among these, the DEX stimulation of GPR91 receptor by an oversecretion of succinate leading to proliferation and survival pathways activation may be a very interesting target for future research.

## Data Availability Statement

The datasets generated for this study are available on request to the corresponding author.

## Author Contributions

MD designed the work, performed the research and draft the manuscript. CS and AD contributed to the acquisition, analysis, and interpretation of data. VT and J-MC revised the work critically for important intellectual content and provided approval for publication of the content.

## Conflict of Interest

The authors declare that the research was conducted in the absence of any commercial or financial relationships that could be construed as a potential conflict of interest.
